# Elucidating the genetic basis of an oligogenic birth defect using whole genome sequence data in a non-model organism, *Bubalus bubalis*

**DOI:** 10.1038/srep39719

**Published:** 2017-01-03

**Authors:** Lynsey K. Whitacre, Jesse L. Hoff, Robert D. Schnabel, Sara Albarella, Francesca Ciotola, Vincenzo Peretti, Francesco Strozzi, Chiara Ferrandi, Luigi Ramunno, Tad S. Sonstegard, John L. Williams, Jeremy F. Taylor, Jared E. Decker

**Affiliations:** 1 Informatics Institute, University of Missouri, Columbia, Missouri, USA; 2 Division of Animal Sciences, University of Missouri, Columbia, Missouri, USA; 3 Department of Veterinary Medicine and Animal Production, University of Naples Federico II, Naples, Italy; 4 Parco Tecnologico Padano, Lodi, Italy; 5 Department of Agriculture, University of Naples Federico II, Portici, Napoli, Italy; 6 Recombinetics, St. Paul, Minnesota, USA; 7 Davies Research Centre, School of Animal and Veterinary Sciences, University of Adelaide, Roseworthy, Australia

## Abstract

Recent strong selection for dairy traits in water buffalo has been associated with higher levels of inbreeding, leading to an increase in the prevalence of genetic diseases such as transverse hemimelia (TH), a congenital developmental abnormality characterized by absence of a variable distal portion of the hindlimbs. Limited genomic resources available for water buffalo required an original approach to identify genetic variants associated with the disease. The genomes of 4 bilateral and 7 unilateral affected cases and 14 controls were sequenced. A concordance analysis of SNPs and INDELs requiring homozygosity unique to all unilateral and bilateral cases revealed two genes, *WNT7A* and *SMARCA4*, known to play a role in embryonic hindlimb development. Additionally, SNP alleles in *NOTCH1* and *RARB* were homozygous exclusively in the bilateral cases, suggesting an oligogenic mode of inheritance. Homozygosity mapping by whole genome *de novo* assembly also supported oligogenic inheritance; implicating 13 genes involved in hindlimb development in bilateral cases and 11 in unilateral cases. A genome-wide association study (GWAS) predicted additional modifier genes. Although our data show a complex inheritance of TH, we predict that homozygous variants in *WNT7A* and *SMARCA4* are necessary for expression of TH and selection against these variants should eradicate TH.

Water buffalo were domesticated approximately 5,000 years ago on the Indian subcontinent^1^. Today, there are over 130 million domesticated water buffalo worldwide that serve as an important component of agriculture through both milk and meat production^2^. In many developing countries, water buffalo account for more than 50% of the milk production and are relied upon more than any other domesticated species^3^,^4^. Recently, a genetic disease called transverse hemimelia (TH) has appeared in Italian Mediterranean River buffalo, most likely as an indirect result of strong selection for dairy production traits and an accompanying increase in the rate of inbreeding. Transverse hemimelia causes unilateral or bilateral hindlimb malformation and is defined by the lack of development of distal hindlimb structures, which manifests as the loss of one or both hindlimbs at a distal point that is variable among cases^5^ (Fig. 1A,B). It was first reported in water buffalo in 2008 after the conclusion of a study of buffalo with limb malformations from farms in the southern Italian region of Campania^6^. In severe cases with bilateral hindlimb malformation, one or both forelimbs may also be affected. Involved limbs appear as to be amputated with the exception of the fact that there are sketches of claws in the terminal part^7^. The prevalence of the disease has been estimated to be between two and five percent in some populations of Mediterranean Italian River buffalo^8^. Unfortunately, because record keeping is poor and pedigrees are often unknown or incomplete, the mode of inheritance of TH in water buffalo has not been established.

Other types of hemimelia, a generalized developmental anomaly resulting in the absence of the distal portions of one or more limbs, have been reported in domestic species including goats, lambs, cattle, dogs, and cats^9^,^10^,^11^,^12^,^13^. In goats, lambs, and cattle, hemimelia has been shown to be heritable, but it can also be caused by environmental exposures to teratogenic plants, parasites, and drugs^14^. The increase in hemimelia in livestock species has been blamed on high levels of inbreeding due to selection for economically valuable traits resulting in increased homozygosity of recessive deleterious mutations. Pedigree analyses of dogs and Shorthorn cattle have revealed hemimelia to be inherited as an autosomal recessive disorder^11^,^12^. Hemimelia has also been reported in humans, but occurs either due to autosomal recessive inheritance or sporadically, suggesting a polygenic mode of inheritance^15^.

Despite several recent research efforts to elucidate the molecular mechanisms involved in hemimelia, the causal mutations in water buffalo and many other species are currently unknown. However, the genetic mechanisms responsible for embryonic hindlimb morphogenesis have been extensively studied in model species and over 30 genes have been implicated in hindlimb development^16^,^17^,^18^,^19^. Furthermore, many genes involved in embryonic morphogenesis have been suggested to play roles in the manifestation and inheritance of TH and could be candidates for the causal mutation^20^,^21^. To elucidate the genes involved in the inheritance of TH in water buffaloes, we sequenced 11 affected buffaloes (4 with bilateral TH and 7 with unilateral TH) and obtained sequences for 14 control buffaloes from the International Water Buffalo Genome Consortium. Our analyses of these data suggest an oligogenic inheritance pattern, and implicate variants in *SMARCA4* and *WNT7A* as the main drivers necessary for the manifestation of TH. The accumulation of homozygous mutations in modifier genes appears to impact the severity of the TH phenotype, resulting in animals that vary from the lack of a single transverse bone in one limb to the complete lack of both hindlimbs with malformation of one forelimb. The analyses leading to these conclusions describe a novel method for detecting the loci underlying an oligogenic disease in a non-model organism that lacks refined genomic resources such as a completed reference genome or annotated gene models.

## Results

### Alignment and variant calling

Alignment of DNA sequences from 11 cases and 14 controls to the UMD_CASPUR_WB_2.0 water buffalo reference assembly resulted in an average mapping rate of 99.17% and average coverage of 9.15X (Supplementary Table 1). Initial variant calling identified approximately 21.7 million SNPs and 2.8 million INDELs. After filtering on quality, 19.8 million SNPs and 2.7 million INDELs remained for analysis. The overall genotype call rate was 98.02% in the cases and 90.99% in the controls; consistent with the reduced depth of sequence coverage and older Illumina chemistry version used to sequence the controls (Supplementary Table 2).

### Case versus control concordance analysis

SNP and INDEL concordance analyses were performed to identify variants for which all cases were homozygous for an allele that was never homozygous in the controls. In total, 1,741 SNPs and 793 INDELs met this criterion (Supplementary Datasets 1, 2). Nine hundred seventy-one of the SNPs were not in genes, but the 770 remaining SNPs were located in 451 unique genes. Two of the genes, *SMARCA4* and *WNT7A*, were associated with the GO term “embryonic hindlimb morphogenesis” (GO:0035116). Furthermore, when only the bilaterally affected cases were analyzed for SNP concordance two additional genes, *NOTCH1* and *RARB*, which are also associated with embryonic hindlimb morphogenesis, were detected. When only the three most severely affected bilateral cases, with the complete loss of both hindlimbs, were analyzed one additional hindlimb morphogenesis related gene, *TFAP2B*, was detected. These findings, along with the fact that no additional associated genes were detected when the unilaterally affected cases were analyzed, present the first genomic evidence for an oligogenic mode of inheritance for TH in water buffalo. However, by aligning the available cattle gene models for these genes to the water buffalo genome assembly, we predicted that all of the disease-associated mutations were located in introns.

Despite the challenge of calling INDEL genotypes with high accuracy from low coverage sequence data, we also analyzed the detected INDELs for their concordance with TH phenotype. Of the 793 concordant INDELs, 222 were located in genes, but only one was found in a gene associated with hindlimb morphogenesis. This INDEL was found in *WNT7A*, a gene also identified by the SNP analysis, and occurs as a 3 bp insertion (C->CCCG). Based on aligning to the *WNT7A* gene annotation in bovine, this variant appears to be located in intron 3. Unlike the concordance analysis performed for SNPs, no additional concordant INDELs were detected as the cases were further restricted according to the severity of the TH phenotype. We interpret the results of the INDEL analysis cautiously because, even after filtering for quality, a large proportion of the remaining INDELs appear to have been identified because of homopolymer repeat errors.

### Homozygosity mapping by de novo assembly

Large runs of homozygosity (ROH) are common in inbred animals, which have an increased risk for genetic diseases because the deleterious effects of recessive alleles are expressed when they are found in a homozygous state^22^. However, with the exception of selective sweep regions that affect all animals, runs of homozygosity should not be shared across large numbers of unrelated individuals. Thus, homozygosity mapping is a powerful method to identify loci responsible for autosomal recessive traits^23^. Assuming a common origin for all cases, ROH should capture the loci that cause TH in distantly related affected animals (see Methods). However, because the water buffalo reference assembly currently exists as an early draft with more than 367,000 unplaced sequence scaffolds, we used a novel approach for homozygosity mapping that was not limited by the reference assembly scaffold lengths.

**Figure 2:**
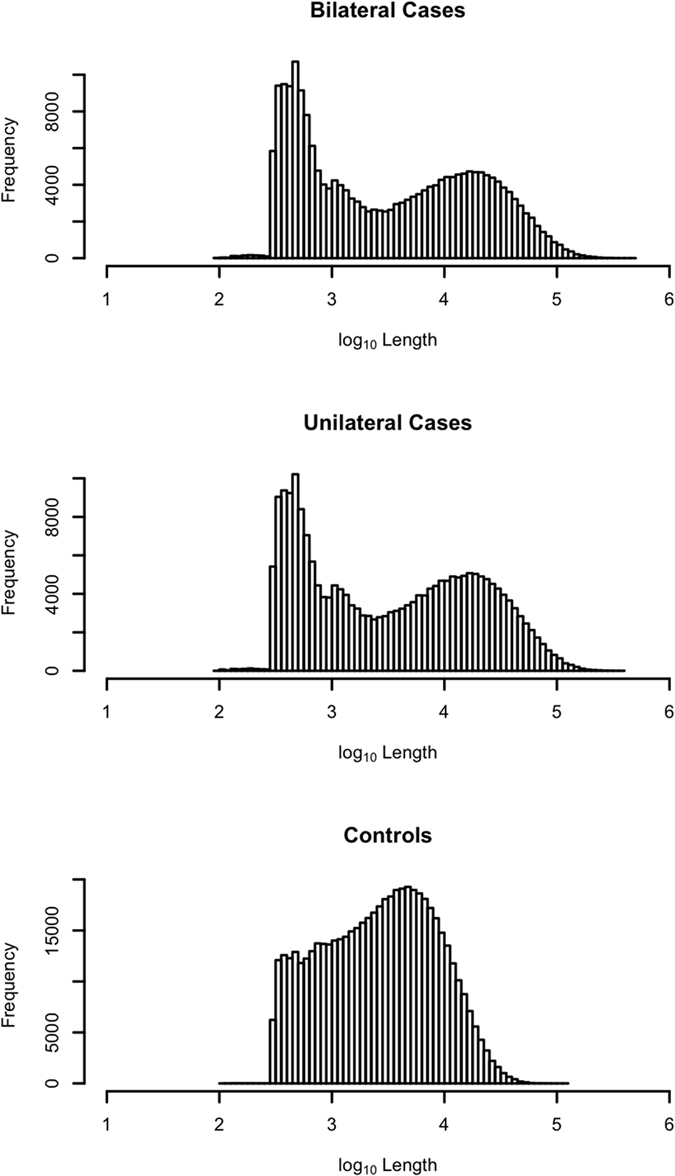
Log_10_-transformed distribution of contig sizes from the *de novo* assembly of pooled sequences from the bilaterally affected cases, unilaterally affected cases and controls.

Three whole genome *de novo* assemblies were performed from the sequence data, which were pooled separately from four bilaterally affected cases, four unilaterally affected cases, and four controls. Regions of the genome with lower heterozygosity in sequence reads pooled from multiple individuals should be assembled into longer contigs, due to reducing forking in the assembly graph. Overall, the contig N50 statistics achieved for the bilaterally affected and unilaterally affected cases were much larger than for the controls (Supplementary Table 3) and contained contigs that were approximately 0.75 orders of magnitude larger than those assembled for the controls (Fig. 2). We were also able to assemble nearly the entire water buffalo genome (~2.64 Gb) in 218,053 contigs in the bilaterally affected cases and in 221,020 contigs in the unilaterally affected cases, both significantly fewer than for the current reference assembly, compared to 541,203 contigs for the controls. Estimated from a negative binomial generalized linear model, the mean contig lengths of the bilateral (12,140.04 bp) and unilateral (11,957.47 bp) assemblies were significantly longer than the mean contig length from the assembly of the control samples (4,738.88 bp), which is most likely due to the higher coverage for these samples (Supplementary Table 3). Further, the mean contig length from the assembly of the bilateral cases was significantly longer than from the assembly of the unilateral cases (Z-score = −4.08, *p*-value = 4.6e–05). Overall, these results clearly indicate an increase in genome-wide homozygosity in the TH affected buffaloes.

We annotated the gene content of the contigs from each assembly that were significantly larger than average by aligning them to the water buffalo reference genome. Following false discovery rate (FDR) correction with q < 0.10, the assembly produced for the controls had 354 contigs significantly larger than the average, the assembly for the unilateral cases had 194 contigs significantly larger than average, and the assembly for the bilateral cases had 365 contigs significantly larger than average. The large contigs identified following FDR correction contained 5, 2, and 0 hindlimb morphogenesis genes for the bilateral cases, the unilateral cases and the controls, respectively (Supplementary Dataset 3). None of these genes were in common with those detected from the SNP and INDEL concordance analyses. However, homozygosity mapping by *de novo* assembly is impacted by the presence of repetitive elements in the genome that are larger than the sequencing library fragment size and terminate contig assembly. The distribution of these elements in the water buffalo genome is unknown as the reference assembly is not of high quality. Therefore, the effect of repetitive elements on disrupting the assembly of long contigs could not be assessed. To compensate for this and include regions that may be largely homozygous but poorly assembled, contigs in the 99^th^ percentile for size from each of the assemblies were also aligned to the water buffalo reference genome to assess their gene content. Analysis of the longest 1% of contigs assembled for the controls, unilaterally and bilaterally affected cases revealed 2, 11, and 13 genes associated with hindlimb morphogenesis, respectively (Supplementary Dataset 4). Six of these genes – *SMARCA4, NOTCH1, CHD7, MSX1, SALL1,* and *TBX3* – were detected in both the bilaterally affected and unilaterally affected cases. The *SMARCA4* locus, which was also identified in the SNP and INDEL analyses, was of particular interest because this gene and its flanking regions were assembled into a single contiguous sequence approximately 140 kb in length in both the bilaterally and unilaterally affected cases, but in the controls was placed on over 40 disjoint contigs (Supplementary Figure 1).

### Genome-wide association study

**Figure 3:**
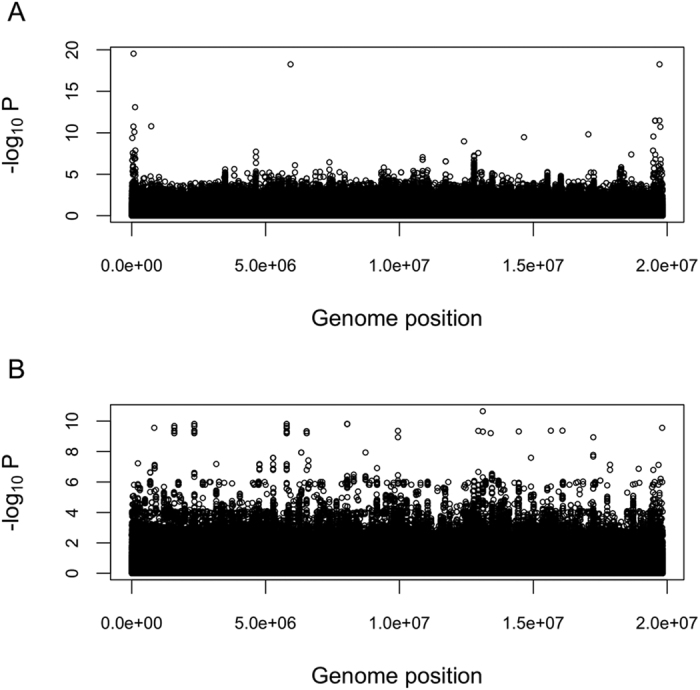
Manhattan plots of GWAS results. (**A**) GWAS results from association with a binary phenotype. (**B**) GWAS results from association with a semi-quantitative phenotype.

Association analyses using both binary and semi-quantitative phenotypes were used to discover additional candidate loci (Fig. 3). Binary phenotypes were simply coded as case *versus* control. Semi-quantitative phenotypes were calculated for each animal based on the number of major distal bones present in each limb ranging from 0 (complete loss of both hindlimbs) to 10 (unaffected control) (Supplementary Table 1). While the binary trait GWAS primarily identified regions on small contigs with no nearby genes, markers near *CHAMP1* and three uncharacterized predicted coding regions exceeded the genome-wide significance threshold (Bonferroni correction) (Supplementary Table 4). *CHAMP1* was also detected by the homozygosity mapping analysis and was on a contig significantly larger than average in both the bilateral and unilateral cases, however, no concordant SNPs or INDELs were identified. GWAS using semi-quantitative phenotypes revealed 15 additional significantly associated genes. These included *FZD4*, a Wnt receptor, and *FGFR1*, a fibroblast growth factor receptor (Supplementary Table 5). The GWAS results also suggested an oligogenic mode of inheritance because numerous loci rose to the same level of significance, in contrast to a GWAS for a Mendelian trait, where one primary peak would be expected in a large sample case *versus* control analysis.

Although the GWAS failed to identify any genes related to hindlimb morphogenesis, several potential modifier genes were detected. This may be due to the nature of the GWAS, which assumed an additive model underlying the severity (expressivity) of the phenotype. The concordance and homozygosity mapping analyses, however, suggested that the phenotype is influenced by epistatic interactions among driver and modifier genes. A further limitation of the GWAS was that SNPs with one or more missing genotypes were either ignored by the analysis algorithm or had association effects estimated by assigning the mean genotype (2 × allele frequency) to missing genotypes. This was particularly problematic here, because of the higher rate of missing genotypes in the controls *versus* cases, due to the lower sequencing depth. Consequently, we filtered results for loci with one or more missing genotypes, resulting in only about 15% of the loci being analyzed for association with the TH phenotypes (Supplementary Table 6).

Although several loci rose to genome-wide significance, we recognize that a larger sample size could provide greater power to detect associated variants. However, it is difficult to estimate the number of samples required to achieve a predetermined power when the trait is oligogenic or polygenic, because the power calculation requires the number of causal loci to be known beforehand. While the mode of inheritance of TH in Italian Mediterranean River buffalo was initially unknown, it was suspected to be a fully penetrant Mendelian defect. With a disease prevalence of ~4%, 11 cases are sufficient to detect a recessive Mendelian disease locus at a significance level of 0.001 with 80% power^24^. While future research will require obtaining and evaluating additional data from affected animals and their parents, we analyzed sequence data for every available case.

### Candidate region mapping

To overcome the challenge of harmonizing significant associations with TH from the variety of performed analyses considering that the water buffalo scaffolds are not assigned to chromosomes, we mapped all of the buffalo genomic regions containing candidate loci to the UMD3.1 bovine reference assembly. While this identified candidate regions on all 29 bovine chromosomes, several chromosomal regions were identified as being significantly associated with TH in all of the performed analyses (Supplementary Figure 2). This again suggests an oligogenic mode of inheritance since loci involved in determining TH are spread throughout the buffalo genome and are not concentrated in a single region as would be expected if TH was inherited as a simple Mendelian.

Rudimentary gene enrichment analyses from the mapping of candidate regions to the bovine reference genome also indicated oligogenicity. From the SNP concordance mapping, a total of 769 candidate genes were discovered. This corresponds to approximately 3.85% of the total number of annotated genes in the genome (n = 19,994; http://useast.ensembl.org/Bos_taurus/Info/Annotation?redirect=no) but 6.06% of the total number of hindlimb morphogenesis genes (n = 31; GO:0035137). When only the bilaterally affected cases were analyzed for SNP concordance, two additional hindlimb morphogenesis genes were detected, but when only the unilaterally affected cases were analyzed no additional genes were associated with the GO term hindlimb morphogenesis. Similarly, from the homozygosity mapping by *de novo* assembly analyses, contigs in the 99^th^ percentile for size from the unilaterally affected cases contained 20.35% of all annotated bovine genes but 33.33% of all hindlimb morphogenesis genes. Large, homozygous contigs assembled from the pooled sequences from the bilaterally affected cases contained 21.36% of all annotated bovine genes but 39.39% of all hindlimb morphogenesis genes. Contigs assembled from the sequences for controls contained 9.70% of all annotated bovine genes but only 6.06% of all hindlimb morphogenesis genes. These results consistently demonstrate an enrichment of hindlimb morphogenesis genes in the larger contigs assembled for the cases compared to the controls with a greater enrichment in the bilaterally versus unilaterally affected cases. They also validate the oligogenic mode of inheritance of TH in Italian Mediterranean River buffalo.

### Gene Ontology Enrichment

Taking into account all 3,988 loci identified as candidates for TH by the various performed analyses, we queried all GO terms to determine sets which may be enriched. The GO term embryonic hindlimb morphogenesis was not significantly enriched (*p*-value > 0.05). However, the collective list of genes identified by SNP concordance, GWAS, and homozygosity mapping was significantly enriched for embryo development (GO:0009795; *p*-value = 0.0163), developmental processes (GO:0032502; *p*-value = 7.04E-9), and anatomical structure development (GO:0048856; *p*-value = 1.57E-9) among others (Supplementary Table 7).

### Network analysis

**Figure 4:**
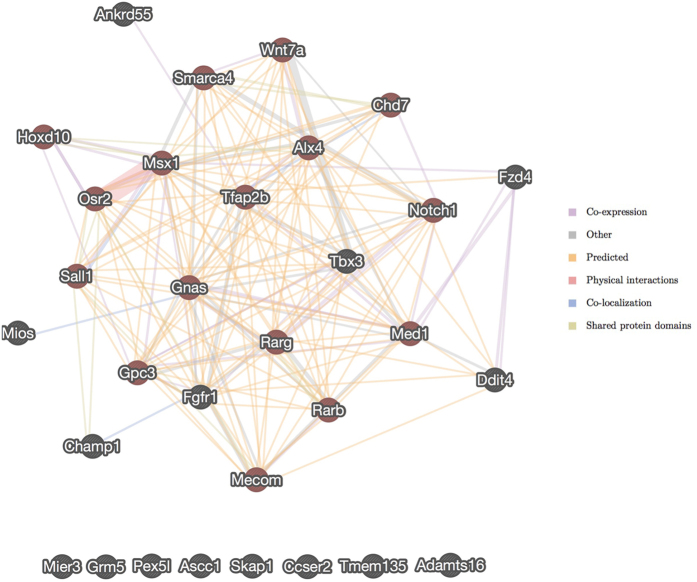
Network analysis of genes predicted to be associated with transverse hemimelia (TH) based on SNP concordance, homozygosity mapping by *de novo* assembly, and GWAS analyses. Genes associated with hindlimb morphogenesis (GO: 0035137) are shaded in red.

Network analysis of all hindlimb morphogenesis genes (n = 16) detected by the SNP concordance and homozygosity mapping analyses and all potential modifier genes identified by the GWAS (n = 15) was performed to understand how these genes may interact. Of these 31 genes, 23 formed an exclusive network based on functional association data including genetic interactions, pathways, co-expression, co-localization, and protein domain similarity (Fig. 4). In this network, *SMARCA4* is directly associated with 12 other genes while *WNT7A* is directly associated with 13 other genes. The remaining 8 genes were not directly associated with any of the other detected genes, and they did not have biological functions that were consistent with the disease phenotype.

## Discussion

We used several tactics to identify the genes involved in TH, a congenital limb abnormality resulting in the loss of transverse elements of the hindlimbs in Italian Mediterranean River buffalo. Although the inheritance pattern of TH was initially unknown, we present evidence for an oligogenic mode of inheritance and identify two primary driver genes and several modifier genes. While mutations in both of the driver genes, *SMARCA4* and *WNT7A*, appear to be necessary for the disease, mutations in the modifier genes contribute to the severity of the expressed phenotype. The *SMARCA4* chromatin remodeling factor is required for normal embryonic development and *SMARCA4* knockouts are lethal^25^,^26^. However, *SMARCA4* expression knockdowns in mice have a large effect on embryonic hindlimb and tail development^19^. These knockdowns produce a phenotype that is very similar to that of the TH affected water buffalo, where the development of the rest of the body and forelimbs is normal and the embryo is viable, but the hindlimbs are extremely underdeveloped.

Several signaling pathways are also required for normal hindlimb development. For example, Wnt signaling has been recognized as important for multiple aspects of mammalian embryonic development^27^, and mutations in *WNT7A* have been reported in hindlimb malformation studies in human and mouse^17^,^28^,^29^. In this research, the identification of two of the three known retinoic acid receptors (*RARG* and *RARB*) from the homozygosity mapping and SNP concordance analyses suggests that the retinoic acid signaling pathways also play a role in embryonic hindlimb development and, subsequently, TH. The role of retinoic acid in limb development is controversial: while previous research has reported an association between hindlimb development and retinoic acid levels^30^,^31^,^32^, a recent study found that hindlimb budding and patterning do not explicitly require retinoic acid signaling^33^. Our data support a role for retinoic acid receptor genes in hindlimb development as the severity of the TH phenotype appears to increase when mutations are also present in these modifier genes. The data also support a role for Notch signaling, as *NOTCH1* was detected both in the SNP concordance analysis and the *de novo* assembly homozygosity mapping. *NOTCH1* and its ligand, *JAG2*, have repeatedly been implicated in hindlimb development^34^,^35^.

We predict that modifier genes interact with *SMARCA4* and *WNT7A* and underlie the oligogenic inheritance pattern and subsequent variable phenotype. This hypothesis is supported by the structure of the network that was generated from the genes identified from the concordance mapping, GWAS, and homozygosity mapping analyses. However, the complex mode of inheritance of TH and the limited data available on both TH affected and control buffaloes preclude identification of the causal mutations, and the molecular mechanism by which the involved genes regulate hindlimb development remains unclear. The variants identified here in *SMARCA4* and *WNT7A* appear necessary for the expression of TH, but are located in introns based on computational predictions. It is possible that these variants are in complete linkage disequilibrium with other variants on the same haplotype that are causal but were not detected in this study, either due to low sequence coverage or gaps in the reference assembly. For example, there are four gaps in *SMARCA4* in the current buffalo reference assembly and there is a large gap just upstream of the gene. The *WNT7A* gene is more completely assembled, but also has one gap within the gene and three gaps in the upstream region. These gaps may contain genomic variants that alter the protein encoded by each gene or that alter gene expression through enhancers, promoters, or transcription factor binding sites. Likewise, the identified intronic variants may themselves disrupt unidentified regulatory elements. Variation in regulatory elements likely contributes to the expression of TH as the down-regulation of the expression of *SMARCA4* produces a similar phenotype in mice^19^.

We predict that selection against the variants found to be homozygous in *SMARCA4* and *WNT7A* in all cases but not in the controls and the avoidance of mating carriers of these variants would quickly eradicate the disease. This hypothesis could be tested by the targeted genotyping of these loci in buffalo affected by TH and their parents to confirm that the loci are reliably predictive of the disease phenotype. The molecular dissection of the effects of mutations in *SMARCA4* and *WNT7A* could then be performed. However, this will likely require the collection of tissues from developing fetuses and possibly also significant improvements in the water buffalo reference genome and its annotation. Nevertheless, we hypothesize that eradication of the disease is now possible by selecting against the disease associated alleles for any of the concordant SNPs detected in *SMARCA4* and/or *WNT7A*.

## Methods

### Sample collection

DNA was collected from 4 bilaterally affected and 7 unilaterally affected TH cases and sequenced on an Illumina HiSeq 2500 at the Parco Tecnologico Padano in Milan, Italy. DNA sequences from 14 control buffaloes were provided by the International Water Buffalo Genome Consortium and were sequenced on an Illumina Genome Analyzer at the USDA Beltsville Research Center, USA. Although the pedigree of all sampled individuals was unknown, a principal component analysis conducted with smartPCA from the EIGENSOFT package^36^ subsequent to variant calling indicated that the cases were not more related to one another than they were to the controls (Supplementary Figure 3). Disease phenotypes were recorded for each case and a semi-quantitative phenotype score was calculated based on the number of major distal bones present in each limb ranging from 0 (complete loss of both hindlimbs) to 10 (unaffected control) (Supplementary Table 1).

### Genome sequencing

All animals were sequenced using Illumina technologies and 2 × 100 bp paired end libraries. Sequence depth varied from 5X to 15X average genome coverage, however, the cases were sequenced to an average depth of 12X and controls to only 7X (Supplementary Table 1). Raw FASTQ sequences have been deposited to NCBI Short Read Archive (SRA) under BioProject PRJNA350833. Supplementary Table 1 contains sample, experiment, and run accessions for each animal.

### Genome alignment and variant detection

Raw sequences were trimmed for adaptors and quality using Trimmomatic-0.33^37^. The reads were then aligned to the UMD_CASPUR_WB_2.0 water buffalo reference assembly (GCF_000471725.1) using the BWA-MEM algorithm, version 0.7.10-r789^38^. Subsequently, we built a variant calling pipeline according to GATK Best Practices and optimized the pipeline for a scaffold level reference genome^39^,^40^,^41^. The pipeline included duplicate removal using Picard (http://broadinstitute.github.io/picard), INDEL realignment, SNP and INDEL discovery using HaplotypeCaller, and genotype calling with GenotypeGVCFs. Base quality score recalibration and variant quality score recalibration were not performed due to the lack of availability of a known reference set of polymorphic sites in water buffalo.

SNP and INDEL variant sites were independently filtered. SNPs were filtered based on the number of detected alleles <3 (biallelic), QD (Variant Confidence/Quality by Depth) <2.0, FS (Phred-scaled *p*-value using Fisher’s exact test to detect strand bias) >60.0, SOR (Symmetric Odds Ratio of 2x2 contingency table to detect strand bias) >4.0, MQ (RMS Mapping Quality) <40.0, MQRankSum (Z-score From Wilcoxon rank sum test of Alt vs. Ref read mapping qualities) <−12.5, or ReadPosRankSum (Z-score from Wilcoxon rank sum test of Alt vs. Ref read position bias) <−8.0. INDELs were filtered based on QD <2.0, FS >200.0, SOR >10.0, ReadPosRankSum <−20.0, or InbreedingCoeff (Inbreeding coefficient as estimated from the genotype likelihoods per-sample when compared against the Hardy-Weinberg expectation) <−0.8. Furthermore, SNPs were filtered on an individual animal basis by setting genotypes with a Phred-scale genotype quality (GQ) <10 to missing.

### Case versus control concordance analysis

Filtered SNPs and INDELs were analyzed for concordance on a case *versus* control basis. This involved sorting variants such that all cases were homozygous for an allele for which none of the controls were homozygous. A missing genotype among the cases caused the variant to be rejected from the analysis, but a missing genotype among the controls was ignored due to the lower mean sequence coverage for the controls.

### Homozygosity mapping by de novo assembly

Three *de novo* genome assemblies were generated: unilaterally affected TH cases, bilaterally affected TH cases, and controls. Each assembly was initiated by pooling sequence reads from four individuals with the respective phenotype. The reads were assembled using MaSuRCA-3.1.3 using default parameters^42^. We used a negative binomial generalized linear model with the glm.nb function from the MASS package^43^ to estimate the mean and dispersion parameter for the contig lengths produced by each assembly. A *p*-value was calculated for each contig to test the hypothesis that the contig was significantly greater in size than the mean, and the *p*-values were corrected for multiple testing by estimating *q*-values^44^. As regions of the genome for which all of the individuals in each pool are homozygous for a single haplotype can be assembled into large contigs (because the assembly graph does not fork), we extracted contigs that were significantly larger than average and those in the 99^th^ percentile for size from each assembly. These contigs were aligned to the UMD_CASPUR_WB_2.0 reference genome assembly and intersected with the water buffalo gene annotation. Finally, the lists of genes in the largest contigs produced from each assembly were compared. Genes within regions that were homozygous in all TH cases, but not in controls, were identified as candidates for risk of TH.

### Genome-wide association study (GWAS)

Given our initial uncertainty as to the mode of inheritance of TH, two GWAS analyses were run. The first was a mixed-model case *versus* control analysis while the second attempted to recover phenotypic information regarding disease severity by scoring the TH phenotypes according to the number of missing hindlimb bones, as previously described. Association tests for both models were performed using univariate linear mixed models and likelihood ratio tests implemented in GEMMA (version 0.94) with a centered genomic relationship matrix^45^. Each analysis was based on 2,990,419 SNPs and statistical significance was determined using a Bonferroni multiple testing correction (*p*-value < 0.05/2990419).

### Candidate region mapping and annotation

Variants identified by the concordance analysis and GWAS were intersected with the water buffalo gene annotation. *De novo* assembled contigs were aligned to the UMD_CASPUR_WB_2.0 water buffalo reference genome assembly using MUMmer3.23^46^. The resulting reference positions were next compared with the water buffalo gene annotation. Additionally, buffalo scaffolds including either a concordant SNP in the case *versus* control analysis or a significant GWAS association after Bonferroni correction and the top 1% of *de novo* contigs were aligned to the *Bos taurus* UMD3.1 reference genome assembly using MUMmer3.23^46^. This allowed us to interpret potential causal loci from the context of a genome as opposed to the 367,000 + unplaced scaffolds.

### Candidate gene ontology and network analysis

Candidate genes identified from SNP concordance analyses, GWAS, and homozygosity mapping were uploaded to the BovineMine warehouse^47^ to compare the list of candidate genes with the list of bovine genes associated with the GO term “hindlimb morphogenesis” (GO: 0035137). Network analyses were performed on the set of candidate genes associated with the GO term “hindlimb morphogenesis” and all candidate genes identified from GWAS using GeneMANIA^48^. We selected only these genes for network analysis because the entire list was too large and because we wanted to use the network analysis to investigate whether genes identified from the GWAS might be acting as modifier genes in conjunction with what we hypothesize to be the primary driver genes. gProfileR version 0.6.1^49^,^50^ was used to conduct GO term enrichment analyses using genes identified from SNP concordance analyses, GWAS from binary and quantitative phenotypes, and homozygosity mapping by *de novo* assembly (99^th^ percentile analysis). A Bonferroni multiple testing correcting and a *p*-value < 0.05 were used to determine statistical significance.

## Additional Information

**Accession codes:** All whole genome sequence data can be found in NCBI SRA under BioProject PRJNA350833. Accession codes can be found in Supplementary Table 1.

**How to cite this article**: Whitacre, L. K. *et al*. Elucidating the genetic basis of an oligogenic birth defect using whole genome sequence data in a non-model organism, *Bubalus bubalis. Sci. Rep.* **7**, 39719; doi: 10.1038/srep39719 (2017).

**Publisher's note:** Springer Nature remains neutral with regard to jurisdictional claims in published maps and institutional affiliations.

## Supplementary Material

Supplementary Information

Supplementary Dataset 1

Supplementary Dataset 2

Supplementary Dataset 3

Supplementary Dataset 4

## Figures and Tables

**Figure 1:**
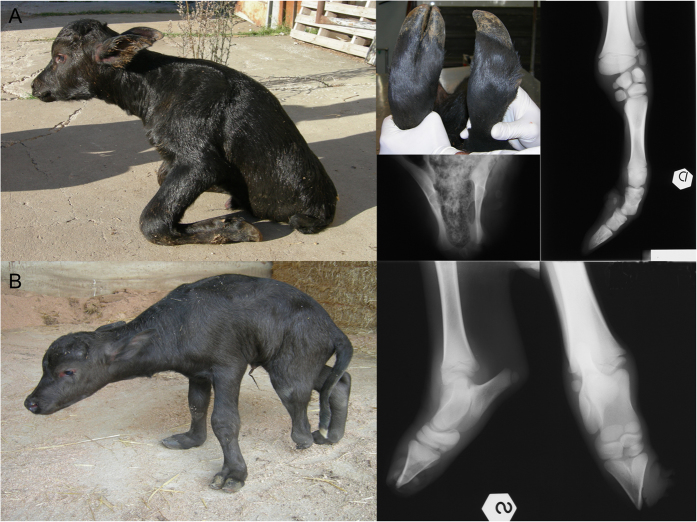
Water buffalo calves with transverse hemimelia (TH). (**A**) A bilaterally affected TH case with both hindlimbs completely absent at birth and hypoplasia of carpal bones and absence of medial bones starting from metacarpus and X-ray images. (**B**) A unilaterally affected TH case with one hindlimb truncated at the tarsus with X-ray image.
