# Supplementary material for: Elucidating the genetic basis of an oligogenic birth defect using whole genome sequence data in a non-model organism, Bubalus bubalis
Source: Sci Rep. 2017 Jan 3;7:39719. doi: 10.1038/srep39719 (PMC5206621; doi:10.1038/srep39719)
Supplement: Supplementary Information [file srep39719-s1.pdf]

Supplementary Information: Elucidating the genetic basis of an oligogenic birth defect using whole genome sequence data in a non-model organism, *Bubalus bubalis*

Lynsey K. Whitacre, Jesse L. Hoff, Robert D. Schnabel, Sara Albarella, Francesca Ciotola, Vincenzo Peretti, Francesco Strozzi, Chiara Ferrandi, Luigi Rammuno, Tad S. Sonstegard, John L. Williams, Jeremy F. Taylor, and Jared E. Decker

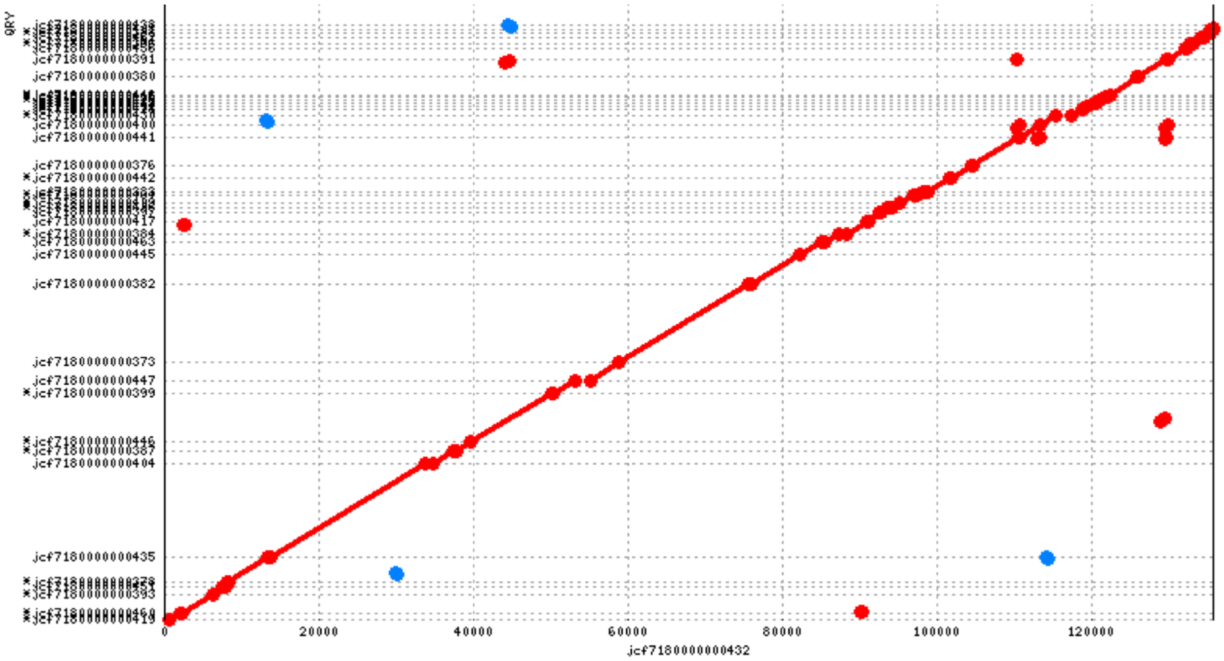

Figure S1. Dot plot of multiple contigs comprising the *SMARCA4* gene region in controls *versus* a single contig comprising the *SMARCA4* gene region in cases indicates increased homozygosity in *SMARCA4* in affected animals.

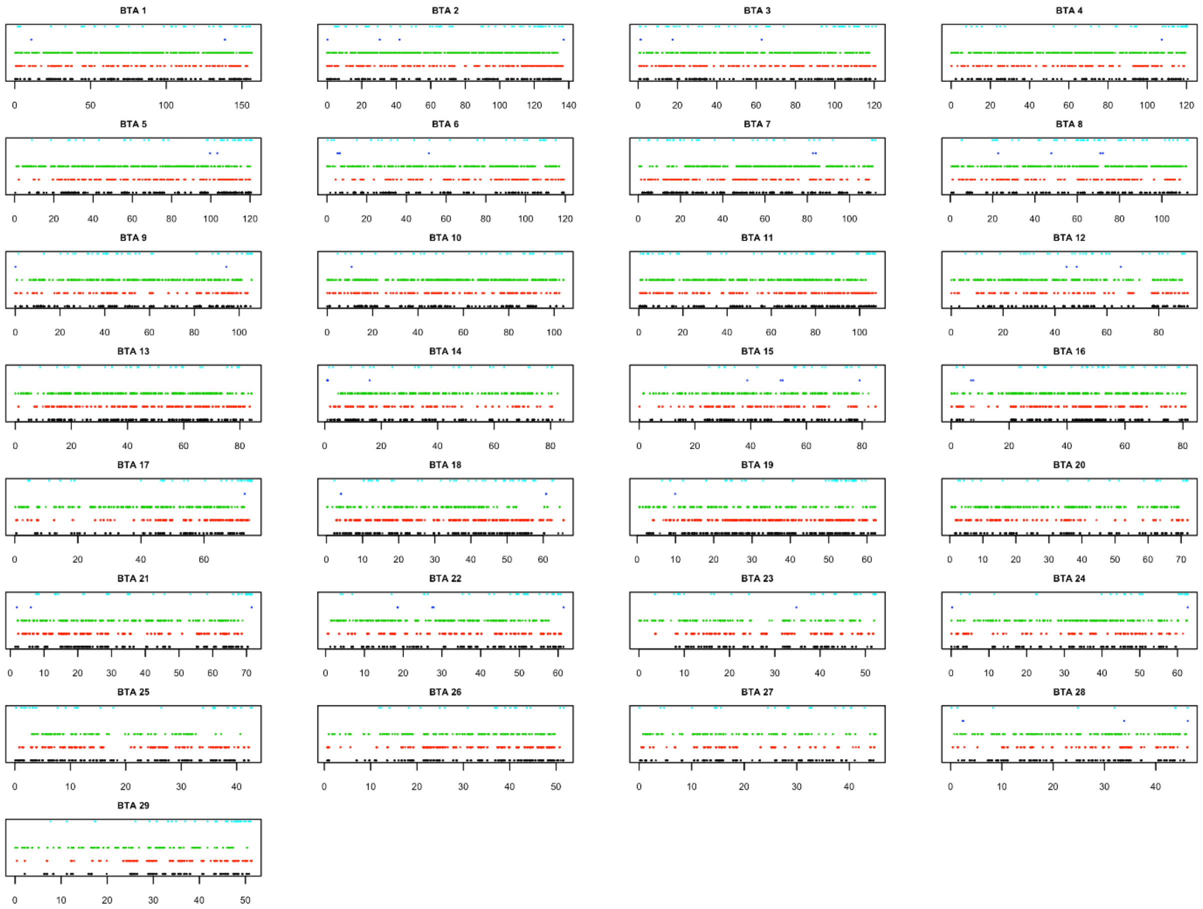

Figure S2. Mapping of regions significantly associated with TH to the *Bos taurus* UMD3.1 reference assembly from bilaterally affected case homozygosity mapping (black), unilaterally affected case homozygosity mapping (red), control homozygosity mapping (green), GWAS (dark blue), and SNP concordance analysis (light blue).

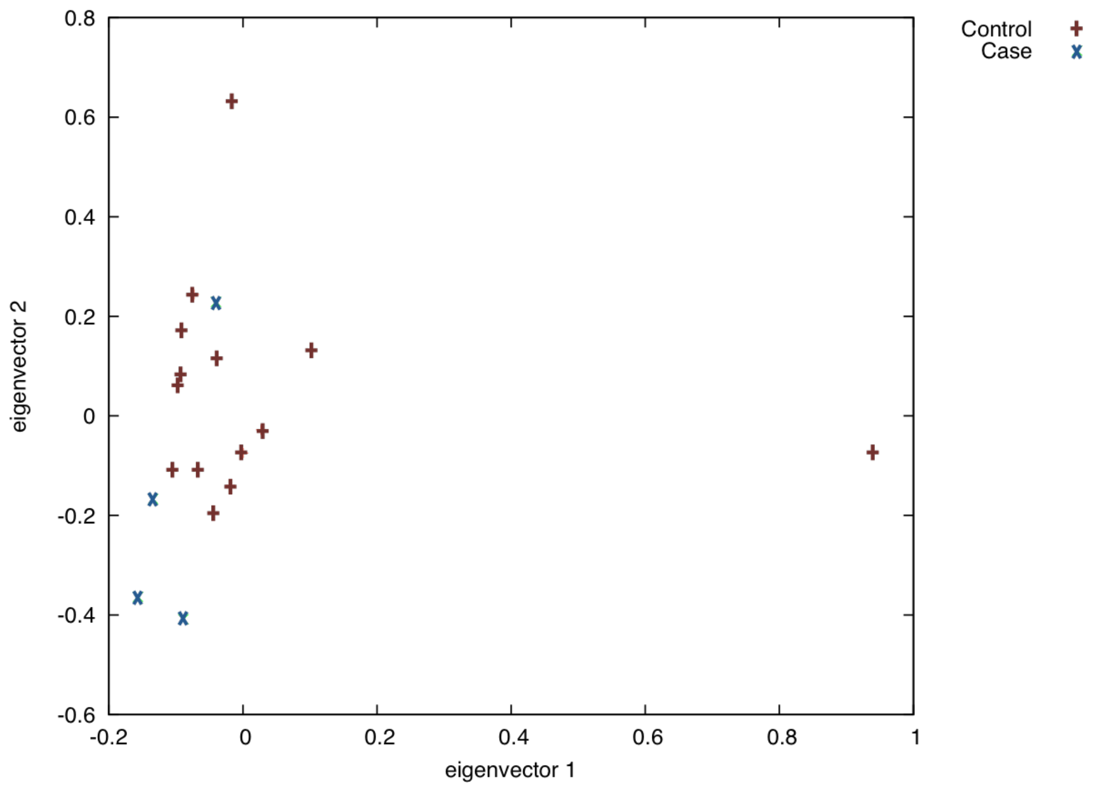

Figure S3. Principal component analysis of genotypes for 11 TH affected cases and 14 controls.

Table S1. Phenotypes and genome sequencing statistics from 11 TH affected cases and 14 unaffected controls.

Sample ID	Total Reads*	Percent Reads Mapped	Percent Mapped with Proper Pairing	Average Genome Coverage	TH Phenotype	TH Phenotype Score	SRA Sample Accession	SRA Experiment Accession	SRA Run Accession
189	472,339,399	99.47	96.08	15.66	Unilateral case (left hindlimb at tarsus)	7	SRS1766349	SRX2281272	SRR4477894
190	249,980,539	98.80	94.47	9.86	Unilateral case (left hindlimb at tarsus)	7	SRS1766350	SRX2281273	SRR4477895
196	395,803,651	99.24	94.74	14.74	Unilateral case (left hindlimb at tarsus)	7	SRS1766351	SRX2281274	SRR4477896
246	269,083,067	98.74	94.44	10.44	Unilateral case (left hindlimb at tarsus)	7	SRS1766352	SRX2281275	SRR4477897
337	318,870,658	98.76	94.85	11.44	Bilateral case (both hindlimbs amputated)	0	SRS1766370	SRX2281293	SRR4477890
581	291,440,073	98.96	94.09	11.23	Unilateral case (left hindlimb at metatarsus)	8	SRS1766353	SRX2281276	SRR4477898
583	348,139,255	98.78	94.49	11.56	Bilateral case (both hindlimbs at fetlock)	8	SRS1766371	SRX2281294	SRR4477891
662	372,645,970	99.26	95.09	14.35	Unilateral case (left hindlimb)	5	SRS1766354	SRX2281277	SRR4477899
701	339,444,712	98.51	94.59	12.37	Bilateral case (both hindlimbs at stifle)	2	SRS1766372	SRX2281295	SRR4477892
850	330,371,536	98.78	94.98	12.32	Bilateral case (both hindlimbs amputated)	0	SRS1766373	SRX2281296	SRR4477893
1095	192,273,051	98.85	94.80	7.63	Unilateral case (left hindlimb at metatarsus)	8	SRS1766355	SRX2281278	SRR4477900
92764	237,146,401	99.53	96.45	7.34	Control	10	SRS1766356	SRX2281279	SRR4477876
92765	293,097,788	99.50	96.40	8.11	Control	10	SRS1766357	SRX2281280	SRR4477877
92766	278,841,904	99.51	96.37	7.92	Control	10	SRS1766358	SRX2281281	SRR4477878
92767	302,482,388	99.56	96.98	8.86	Control	10	SRS1766359	SRX2281282	SRR4477879
92768	235,770,765	99.57	95.60	7.43	Control	10	SRS1766360	SRX2281283	SRR4477880
92769	198,150,064	98.38	95.11	3.28	Control	10	SRS1766361	SRX2281284	SRR4477881
92770	290,662,225	99.61	97.35	9.02	Control	10	SRS1766362	SRX2281285	SRR4477882
92771	252,165,270	99.62	97.36	8.45	Control	10	SRS1766363	SRX2281286	SRR4477883
92774	328,856,248	99.44	90.37	6.66	Control	10	SRS1766364	SRX2281287	SRR4477884

92775	339,570,423	99.14	87.01	6.63	Control	10	SRS1766365	SRX2281288	SRR4477885
92776	326,975,621	99.36	90.76	6.54	Control	10	SRS1766366	SRX2281289	SRR4477886
92777	281,036,266	98.72	85.69	4.57	Control	10	SRS1766367	SRX2281290	SRR4477887
92778	326,105,820	99.53	93.71	7.47	Control	10	SRS1766368	SRX2281291	SRR4477888
92795	151,865,510	99.54	84.69	4.80	Control	10	SRS1766369	SRX2281292	SRR4477889
Average	296,924,744	99.17	93.86	9.15					

*After quality filtering

Table S2. Variant call rates for cases and controls.

Sample ID	Missing Genotypes	Total Genotypes	Frequency of Missing
1095	705,137	19,829,264	0.0356
189	318,869	19,829,264	0.0161
190	483,836	19,829,264	0.0244
196	320,074	19,829,264	0.0161
246	428,965	19,829,264	0.0216
337	361,217	19,829,264	0.0182
581	385,264	19,829,264	0.0194
583	341,047	19,829,264	0.0172
662	287,239	19,829,264	0.0145
701	329,280	19,829,264	0.0166
850	347,345	19,829,264	0.0175
92764	1,293,640	19,829,264	0.0652
92765	1,318,679	19,829,264	0.0665
92766	1,204,282	19,829,264	0.0607
92767	943,547	19,829,264	0.0476
92768	1,161,960	19,829,264	0.0586
92769	5,517,056	19,829,264	0.2782
92770	945,464	19,829,264	0.0477
92771	883,420	19,829,264	0.0446
92774	1,726,724	19,829,264	0.0871
92775	1,787,533	19,829,264	0.0902
92776	1,793,981	19,829,264	0.0905
92777	2,860,236	19,829,264	0.1442
92778	1,388,107	19,829,264	0.0700
92795	2,196,779	19,829,264	0.1108
Case Mean			0.0198
Control Mean			0.0901
Total Mean			0.0592

Table S3. *De novo* assembly statistics from pools of genome sequence data for four individuals.

Sample Group	Pooled Coverage	Total Bases in Scaffolds	Number of Scaffolds	Scaffold N50
Bilateral cases	47.69	2,645,914,807	218,053	36,594
Unilateral cases	50.70	2,641,090,365	221,020	34,411
Control	31.96	2,563,943,329	541,203	8,898

Table S4. Significant GWAS associations under the binary phenotype model.

Contig	Position	P-value	*Candidate gene(s)*
gi|551741020|ref|NW_005765473.1|	1041	4.18E-10	--
gi|551733415|ref|NW_005773077.1|	1806	1.76E-11	--
gi|551732811|ref|NW_005773470.1|	858	2.88E-20	--
gi|551731300|ref|NW_005774981.1|	6595	8.05E-11	--
gi|551730022|ref|NW_005776259.1|	1637	8.16E-14	--
gi|551729656|ref|NW_005776625.1|	4632	1.35E-08	--
gi|551723571|ref|NW_005782710.1|	3036	1.63E-11	--
gi|551721651|ref|NW_005784628.1|	324709	5.55E-19	*LOC102406836*
gi|551720955|ref|NW_005785322.1|	62402	1.09E-09	*LOC102402736, LOC102402095*
gi|551720771|ref|NW_005785506.1|	71732	3.45E-10	--
gi|551720570|ref|NW_005785701.1|	458656	1.54E-10	*CHAMP1*
gi|552101018|ref|NW_005438356.1|	524	1.39E-08	--
gi|552093047|ref|NW_005445879.1|	2664	2.80E-10	--
gi|552029652|ref|NW_005499085.1|	592	3.56E-12	--
gi|552021103|ref|NW_005507230.1|	1062	3.32E-12	--
gi|551818273|ref|NW_005688295.1|	6134	3.32E-12	--
gi|551818114|ref|NW_005688454.1|	2244	5.55E-19	--
gi|551814314|ref|NW_005692254.1|	522	1.91E-11	--

Table S5. Significant GWAS associations under the semi-quantitative phenotype model.

Contig	Position	P-value	Candidate gene(s)
gi|551723235|ref|NW_005783046.1|	2615	2.75E-10	--
gi|551722604|ref|NW_005783677.1|	1032062	4.23E-10	--
gi|551722604|ref|NW_005783677.1|	1032137	6.23E-10	--
gi|551722604|ref|NW_005783677.1|	1044302	2.75E-10	--
gi|551722604|ref|NW_005783677.1|	1050027	6.23E-10	--
gi|551722604|ref|NW_005783677.1|	1078885	4.68E-10	--
gi|551722604|ref|NW_005783677.1|	1079152	2.09E-10	--
gi|551722330|ref|NW_005783951.1|	288101	1.54E-10	*ADAMTS16*
gi|551722330|ref|NW_005783951.1|	311320	4.84E-10	*ADAMTS16*
gi|551722330|ref|NW_005783951.1|	323554	4.68E-10	*ADAMTS16*
gi|551722330|ref|NW_005783951.1|	326651	4.23E-10	*ADAMTS16*
gi|551722330|ref|NW_005783951.1|	337775	1.54E-10	*ADAMTS16*
gi|551722330|ref|NW_005783951.1|	340646	2.09E-10	*ADAMTS16*
gi|551722330|ref|NW_005783951.1|	356823	4.45E-10	*ADAMTS16*
gi|551722330|ref|NW_005783951.1|	358391	6.23E-10	*ADAMTS16*
gi|551722330|ref|NW_005783951.1|	358393	6.23E-10	*ADAMTS16*
gi|551722330|ref|NW_005783951.1|	358958	2.09E-10	*ADAMTS16*
gi|551722330|ref|NW_005783951.1|	359017	4.26E-10	*ADAMTS16*
gi|551722330|ref|NW_005783951.1|	359399	2.09E-10	*ADAMTS16*
gi|551722330|ref|NW_005783951.1|	361386	4.64E-10	*ADAMTS16*
gi|551722330|ref|NW_005783951.1|	362978	4.68E-10	*ADAMTS16*
gi|551722330|ref|NW_005783951.1|	363061	4.93E-10	*ADAMTS16*
gi|551722330|ref|NW_005783951.1|	363067	4.93E-10	*ADAMTS16*
gi|551721677|ref|NW_005784602.1|	9999	2.09E-10	--
gi|551721677|ref|NW_005784602.1|	59585	1.54E-10	*PEX5L*
gi|551721677|ref|NW_005784602.1|	59586	1.54E-10	*PEX5L*
gi|551721677|ref|NW_005784602.1|	59627	6.23E-10	*PEX5L*
gi|551721677|ref|NW_005784602.1|	76344	6.23E-10	*PEX5L*
gi|551721677|ref|NW_005784602.1|	83868	4.68E-10	*PEX5L*
gi|551721677|ref|NW_005784602.1|	84207	2.75E-10	*PEX5L*

gi|551721677|ref|NW_005784602.1|	92660	5.01E-10	*PEX5L*
gi|551721677|ref|NW_005784602.1|	94269	4.93E-10	*PEX5L*
gi|551721677|ref|NW_005784602.1|	94588	6.23E-10	*PEX5L*
gi|551721677|ref|NW_005784602.1|	94796	4.93E-10	*PEX5L*
gi|551721677|ref|NW_005784602.1|	94970	5.04E-10	*PEX5L*
gi|551721677|ref|NW_005784602.1|	95368	6.23E-10	*PEX5L*
gi|551721677|ref|NW_005784602.1|	95504	6.23E-10	*PEX5L*
gi|551721677|ref|NW_005784602.1|	95648	2.75E-10	*PEX5L*
gi|551721677|ref|NW_005784602.1|	95764	2.75E-10	*PEX5L*
gi|551721597|ref|NW_005784682.1|	930485	1.16E-08	--
gi|551721597|ref|NW_005784682.1|	944705	1.16E-08	--
gi|551721575|ref|NW_005784704.1|	540038	4.68E-10	*MIER3*
gi|551721575|ref|NW_005784704.1|	547826	4.68E-10	*MIER3*
gi|551721575|ref|NW_005784704.1|	551466	4.68E-10	*MIER3*
gi|551721575|ref|NW_005784704.1|	553129	4.68E-10	*MIER3*
gi|551721575|ref|NW_005784704.1|	1161864	6.23E-10	*ANKRD55*
gi|551721575|ref|NW_005784704.1|	1161916	6.23E-10	*ANKRD55*
gi|551721409|ref|NW_005784868.1|	68605	1.54E-10	--
gi|551721409|ref|NW_005784868.1|	96621	1.54E-10	--
gi|551721409|ref|NW_005784868.1|	107282	1.54E-10	--
gi|551721409|ref|NW_005784868.1|	107294	1.54E-10	--
gi|551721409|ref|NW_005784868.1|	130154	1.54E-10	--
gi|551721409|ref|NW_005784868.1|	163189	1.54E-10	--
gi|551721329|ref|NW_005784948.1|	2011086	1.15E-08	*MIOS*
gi|551721209|ref|NW_005785068.1|	5901500	4.36E-10	*ASCC1*
gi|551721209|ref|NW_005785068.1|	5901586	1.15E-09	*ASSC1*
gi|551720917|ref|NW_005785360.1|	27661	4.40E-10	*LOC102400491*
gi|551720904|ref|NW_005785373.1|	6985	2.25E-11	*FZD4*
gi|551720904|ref|NW_005785373.1|	572932	4.95E-10	*TMEM135, FZD4*
gi|551720881|ref|NW_005785396.1|	1623273	6.23E-10	*FGFR1*
gi|551720789|ref|NW_005785488.1|	1398022	4.80E-10	*CCSER2*
gi|551720688|ref|NW_005785589.1|	1335886	4.26E-10	*SKAP1*
gi|551720649|ref|NW_005785623.1|	1084940	4.23E-10	*GRM5*

gi|551720521|ref|NW_005785747.1|	19920	1.66E-08	*LOC102412685*
gi|551720521|ref|NW_005785747.1|	427178	1.15E-09	*DDIT4*
gi|551767899|ref|NW_005738669.1|	1277	2.75E-10	--

Table S6. Model statistics from binary and semi-quantitative GWAS.

	Binary Phenotype	Quantitative Phenotype
Total SNPs	19,829,264	19,829,264
Analyzed SNPs	2,990,419	2,990,419
REMLE log-likelihood	-16.769	-61.328
MLE log-likelihood	-11.789	-57.888
pve estimate	1.000	1.000E-05
se(pve)	0.005	-nan
vg estimate	0.767	9.707E-05
ve estimate	7.674E-06	9.707
beta estimate	0.440	7.960
se(beta)	0.001	0.623

Table S11. Significant results from gene ontology enrichment analysis with gProfileR.

GO Term	GO ID	P-value
protein binding	GO:0005515	6.75E-14
intracellular part	GO:0044424	7.64E-13
intracellular	GO:0005622	3.58E-12
system development	GO:0048731	8.54E-12
cytoplasm	GO:0005737	1.59E-11
single-organism process	GO:0044699	7.74E-11
multicellular organism development	GO:0007275	9.95E-11
positive regulation of biological process	GO:0048518	1.70E-10
positive regulation of cellular process	GO:0048522	1.78E-10
cellular process	GO:0009987	5.93E-10
single-organism cellular process	GO:0044763	6.86E-10
positive regulation of metabolic process	GO:0009893	1.44E-09
anatomical structure development	GO:0048856	1.57E-09
cytosol	GO:0005829	1.59E-09
cellular component organization or biogenesis	GO:0071840	2.44E-09
nervous system development	GO:0007399	4.67E-09
positive regulation of gene expression	GO:0010628	5.11E-09
cell differentiation	GO:0030154	6.94E-09
developmental process	GO:0032502	7.04E-09
single-organism developmental process	GO:0044767	7.67E-09
animal organ development	GO:0048513	9.89E-09
binding	GO:0005488	1.09E-08
cellular component organization	GO:0016043	1.17E-08
positive regulation of macromolecule metabolic process	GO:0010604	1.19E-08
cytoplasmic part	GO:0044444	2.10E-08
neurogenesis	GO:0022008	2.22E-08
organelle	GO:0043226	2.45E-08
negative regulation of biological process	GO:0048519	2.58E-08
positive regulation of nitrogen compound metabolic process	GO:0051173	5.77E-08
regulation of transcription from RNA polymerase II promoter	GO:0006357	6.38E-08

nervous system development	GO:0007399	8.45E-08
positive regulation of macromolecule biosynthetic process	GO:0010557	8.45E-08
generation of neurons	GO:0048699	1.07E-07
negative regulation of cellular process	GO:0048523	1.20E-07
regulation of signaling	GO:0023051	1.53E-07
single-multicellular organism process	GO:0044707	2.38E-07
cellular developmental process	GO:0048869	2.39E-07
regulation of cell communication	GO:0010646	3.43E-07
programmed cell death	GO:0012501	4.15E-07
anatomical structure morphogenesis	GO:0009653	4.26E-07
neuron differentiation	GO:0030182	5.08E-07
positive regulation of nucleobase-containing compound metabolic process	GO:0045935	5.94E-07
positive regulation of RNA metabolic process	GO:0051254	7.24E-07
intracellular organelle	GO:0043229	7.43E-07
transcription from RNA polymerase II promoter	GO:0006366	8.23E-07
organelle part	GO:0044422	8.80E-07
positive regulation of cellular metabolic process	GO:0031325	9.08E-07
positive regulation of transcription, DNA-templated	GO:0045893	1.00E-06
positive regulation of nucleic acid-templated transcription	GO:1903508	1.00E-06
positive regulation of biosynthetic process	GO:0009891	1.09E-06
positive regulation of RNA biosynthetic process	GO:1902680	1.17E-06
membrane-bounded organelle	GO:0043227	1.33E-06
intracellular membrane-bounded organelle	GO:0043231	1.33E-06
intracellular organelle part	GO:0044446	1.64E-06
localization	GO:0051179	1.67E-06
regulation of signal transduction	GO:0009966	1.80E-06
biological_process	GO:0008150	2.32E-06
positive regulation of cellular biosynthetic process	GO:0031328	2.36E-06
positive regulation of transcription from RNA polymerase II promoter	GO:0045944	2.38E-06
cell death	GO:0008219	2.48E-06
apoptotic process	GO:0006915	2.71E-06
cellular macromolecule metabolic process	GO:0044260	3.84E-06
metabolic process	GO:0008152	8.71E-06

cell	GO:0005623	9.74E-06
establishment of localization in cell	GO:0051649	1.06E-05
cell development	GO:0048468	1.27E-05
nuclear part	GO:0044428	1.82E-05
regulation of molecular function	GO:0065009	2.17E-05
cell part	GO:0044464	2.41E-05
cell morphogenesis involved in differentiation	GO:0000904	2.58E-05
establishment of localization	GO:0051234	2.84E-05
single-organism localization	GO:1902578	2.93E-05
cellular component organization	GO:0016043	3.00E-05
localization	GO:0051179	3.42E-05
cellular localization	GO:0051641	3.48E-05
neuron part	GO:0097458	3.48E-05
cellular metabolic process	GO:0044237	3.68E-05
anatomical structure development	GO:0048856	4.03E-05
regulation of cell death	GO:0010941	4.25E-05
cell development	GO:0048468	5.26E-05
macromolecule metabolic process	GO:0043170	6.37E-05
transcription factor binding	GO:0008134	0.000118
regulation of programmed cell death	GO:0043067	0.000119
cellular_component	GO:0005575	0.000122
multicellular organism development	GO:0007275	0.000156
cellular component organization or biogenesis	GO:0071840	0.000156
regulation of response to stimulus	GO:0048583	0.00018
generation of neurons	GO:0048699	0.000189
single-organism transport	GO:0044765	0.00021
cellular component assembly	GO:0022607	0.000295
cellular protein metabolic process	GO:0044267	0.000297
nuclear lumen	GO:0031981	0.00034
cell projection	GO:0042995	0.000353
nucleoplasm	GO:0005654	0.000361
axon development	GO:0061564	0.000378
regulation of catalytic activity	GO:0050790	0.000391

regulation of metabolic process	GO:0019222	0.000393
regulation of apoptotic process	GO:0042981	0.000416
intracellular signal transduction	GO:0035556	0.000426
neuron part	GO:0097458	0.000456
macromolecular complex binding	GO:0044877	0.000476
neurogenesis	GO:0022008	0.000477
regulation of cellular component organization	GO:0051128	0.000483
transport	GO:0006810	0.000485
system development	GO:0048731	0.000527
sequence-specific DNA binding	GO:0043565	0.00053
cell surface receptor signaling pathway	GO:0007166	0.000532
regulation of developmental process	GO:0050793	0.000551
organic substance metabolic process	GO:0071704	0.000551
protein binding	GO:0005515	0.00061
regulation of macromolecule metabolic process	GO:0060255	0.000642
intracellular organelle lumen	GO:0070013	0.000672
regulation of intracellular signal transduction	GO:1902531	0.000787
central nervous system development	GO:0007417	0.000838
synapse	GO:0045202	0.000866
single-organism cellular process	GO:0044763	0.000877
movement of cell or subcellular component	GO:0006928	0.000892
axonogenesis	GO:0007409	0.000903
regulation of multicellular organismal development	GO:2000026	0.000934
synaptic transmission	GO:0007268	0.000961
synaptic signaling	GO:0099536	0.000961
trans-synaptic signaling	GO:0099537	0.000961
cell junction	GO:0030054	0.000992
cell morphogenesis	GO:0000902	0.001
cell-cell signaling	GO:0007267	0.00107
organelle lumen	GO:0043233	0.0011
cellular component morphogenesis	GO:0032989	0.00113
axon guidance	GO:0007411	0.00135
neuron projection guidance	GO:0097485	0.00135

positive regulation of biological process	GO:0048518	0.0017
neuron development	GO:0048666	0.00172
cytoplasm	GO:0005737	0.00173
developmental process	GO:0032502	0.002
cytoplasmic part	GO:0044444	0.00205
cell projection	GO:0042995	0.00217
single-organism developmental process	GO:0044767	0.00219
neuron differentiation	GO:0030182	0.00223
regulation of multicellular organismal process	GO:0051239	0.00228
cellular nitrogen compound biosynthetic process	GO:0044271	0.00238
regulatory region DNA binding	GO:0000975	0.00239
RNA polymerase II transcription factor activity, sequence-specific DNA binding	GO:0000981	0.0024
membrane-enclosed lumen	GO:0031974	0.00246
neuron development	GO:0048666	0.0025
regulatory region nucleic acid binding	GO:0001067	0.00256
protein complex	GO:0043234	0.00303
transcription regulatory region DNA binding	GO:0044212	0.00343
regulation of localization	GO:0032879	0.00353
sensory organ development	GO:0007423	0.00371
neuron projection	GO:0043005	0.00391
regulation of nervous system development	GO:0051960	0.00423
binding	GO:0005488	0.00435
regulation of cell development	GO:0060284	0.00435
response to organic substance	GO:0010033	0.00441
nucleus	GO:0005634	0.00445
tube morphogenesis	GO:0035239	0.00456
single-organism process	GO:0044699	0.00479
positive regulation of cellular process	GO:0048522	0.00484
movement of cell or subcellular component	GO:0006928	0.00507
negative regulation of macromolecule metabolic process	GO:0010605	0.0052
vesicle-mediated transport	GO:0016192	0.0054
presynaptic process involved in synaptic transmission	GO:0099531	0.00554
protein metabolic process	GO:0019538	0.00569

regulation of cell differentiation	GO:0045595	0.00574
regulation of hydrolase activity	GO:0051336	0.006
cell morphogenesis involved in neuron differentiation	GO:0048667	0.00616
regulation of cell proliferation	GO:0042127	0.00631
regulation of transport	GO:0051049	0.00647
primary metabolic process	GO:0044238	0.00658
tissue development	GO:0009888	0.00672
cellular response to organic substance	GO:0071310	0.00675
neuron projection morphogenesis	GO:0048812	0.00712
double-stranded DNA binding	GO:0003690	0.00714
cellular component biogenesis	GO:0044085	0.00823
neuron projection development	GO:0031175	0.00844
signaling	GO:0023052	0.00856
cell projection organization	GO:0030030	0.00969
single-multicellular organism process	GO:0044707	0.00969
neuron projection	GO:0043005	0.00999
endomembrane system organization	GO:0010256	0.0102
regulation of cellular protein metabolic process	GO:0032268	0.011
intracellular part	GO:0044424	0.0113
gene expression	GO:0010467	0.0117
actin filament-based process	GO:0030029	0.0118
regulation of neurotransmitter levels	GO:0001505	0.0121
actin cytoskeleton organization	GO:0030036	0.0128
secretion	GO:0046903	0.0128
signal release	GO:0023061	0.013
single organism signaling	GO:0044700	0.013
organ morphogenesis	GO:0009887	0.0135
regulation of localization	GO:0032879	0.0137
organelle organization	GO:0006996	0.0138
positive regulation of cellular component organization	GO:0051130	0.0139
regulation of cellular metabolic process	GO:0031323	0.0144
cell communication	GO:0007154	0.0146
negative regulation of cellular metabolic process	GO:0031324	0.0147

organelle localization	GO:0051640	0.0147
neurotransmitter transport	GO:0006836	0.015
chemotaxis	GO:0006935	0.0153
cell proliferation	GO:0008283	0.0154
taxis	GO:0042330	0.0162
embryo development	GO:0009790	0.0163
signal release from synapse	GO:0099643	0.0164
neuron projection development	GO:0031175	0.0165
neurotransmitter secretion	GO:0007269	0.0178
regulation of protein metabolic process	GO:0051246	0.0182
multicellular organismal process	GO:0032501	0.0188
negative regulation of metabolic process	GO:0009892	0.019
tube development	GO:0035295	0.0196
cell projection organization	GO:0030030	0.0199
positive regulation of molecular function	GO:0044093	0.0199
regulation of transmembrane transport	GO:0034762	0.0214
negative regulation of signaling	GO:0023057	0.0221
cell differentiation	GO:0030154	0.0242
sequence-specific double-stranded DNA binding	GO:1990837	0.0246
negative regulation of protein modification process	GO:0031400	0.0253
synapse part	GO:0044456	0.0256
regulation of cellular process	GO:0050794	0.0285
transcription regulatory region sequence-specific DNA binding	GO:0000976	0.0286
nitrogen compound metabolic process	GO:0006807	0.0299
macromolecular complex	GO:0032991	0.0303
regulation of anatomical structure morphogenesis	GO:0022603	0.0308
macromolecular complex	GO:0032991	0.0341
cellular nitrogen compound metabolic process	GO:0034641	0.0357
positive regulation of cellular component organization	GO:0051130	0.0368
regulation of cellular component organization	GO:0051128	0.0382
locomotion	GO:0040011	0.0384
cell	GO:0005623	0.0406
negative regulation of transcription from RNA polymerase II promoter	GO:0000122	0.0418

negative regulation of cell communication	GO:0010648	0.0422
regulation of nervous system development	GO:0051960	0.0422
cytoskeleton organization	GO:0007010	0.0438
regulation of neurogenesis	GO:0050767	0.0439
regulation of cell development	GO:0060284	0.0449
cellular protein modification process	GO:0006464	0.05
peroxisome organization	GO:0007031	0.05
protein localization	GO:0008104	0.05
protein modification process	GO:0036211	0.05
